# Inhibitory effect of aloperine on transient outward potassium currents in rat cardiac myocytes

**DOI:** 10.3389/fphar.2024.1372973

**Published:** 2024-03-28

**Authors:** Xiao-Na Dong, Meng-Ting Li

**Affiliations:** ^1^ Clinical Medical College, Yangzhou University, Yangzhou, China; ^2^ Baoying County Traditional Chinese Medicine Hospital, Yangzhou, China

**Keywords:** aloperine, transient outward potassium current, whole-cell patch-clamp, atrial fibrillation, molecular docking

## Abstract

**Objective:**

Aloperine (ALO) is an effective quinolizidine alkaloid. Previous research has demonstrated its antiarrhythmic effect by inhibiting voltage-gated sodium currents in rat ventricular myocytes. This study explored its effect on transient outward potassium currents (I_to_) in rat atrial myocytes to identify potential targets in the context of ion channel currents.

**Methods:**

The I_to_ characteristics in rat atrial myocytes were recorded using a whole-cell patch-clamp technique. Molecular docking was performed to validate ligand-protein binding interactions.

**Results:**

ALO at concentrations of 3 and 10 μM significantly reduced I_to_ current densities. Gating kinetics analysis revealed ALO’s ability to slow I_to_ activation, hasten inactivation, and prolong transition from inactive to resting state. Molecular docking revealed that ALO could stably bind to *KCND2*.

**Conclusion:**

ALO may inhibit I_to_ by slowing the activation process, accelerating inactivation, and delaying the recovery time after inactivation, potentially preventing acetylcholine-induced AF.

## 1 Introduction

Cardiac arrhythmias refer to any deviation from the normal heart rhythm. Atrial fibrillation (AF) is a global health problem, and its significant impact on disability and mortality has made it a focal point of research in the field of cardiac arrhythmias. Animal models are key in the study of AF pathogenesis, which can be similarized to AF in humans with similar natural onset of the disease.

Recently, monomeric compounds extracted from traditional Chinese herbal medicines for the treatment of cardiovascular diseases have attracted substantial attention due to their high efficiency and safety (Xu and Yang, 2009). *Sophora alopecuroides* L., a perennial shrub, harbors several active components such as quinolizidine alkaloids, flavonoids, and flavone stilbenes. These compounds have shown efficacy in treating acute dysentery and ameliorating specific cardiovascular conditions (Li Y. et al., 2020; Wang et al., 2020; Zhou et al., 2020). Among these compounds, aloperine (ALO), a quinolizidine alkaloid, has gained attention from researchers worldwide (Wang et al., 2015; Lv et al., 2020; Ye et al., 2020). Studies have revealed ALO’s potential in enhancing cardiac function, particularly in conditions such as ischemia-reperfusion and coronary artery microembolization (Mao et al., 2019). Moreover, ALO can reduce lipid peroxidation and ventricular remodeling in rats with atherosclerosis-induced myocardial hypertrophy (Li W. et al., 2020).

Evidence suggests that ion channels of cardiac myocytes are key to understanding the mechanisms of cardiac dysfunction and arrhythmia (Thiel et al., 1997). Previous research has demonstrated antiarrhythmic effect of ALO by inhibiting voltage-gated sodium currents in rat ventricular myocytes in a concentration-dependent manner (Li et al., 2021). Voltage-dependent potassium (Kv) channels play crucial role in repolarizing cell membranes and modulating membrane excitability, with various subtypes in the heart that are associated with diseases (MacKinnon, 1995). Among these, Kv4.2 (encoded by *KCND2*) and Kv4.3 (encoded by *KCND3*) act as pore-forming subunits responsible for generating the transient outward K^+^ current (I_to_) (Drum et al., 2016; Zhang et al., 2021). I_to_ constitutes a six transmembrane segmented (S1–S6) ion channel involved in mediating the transient outward K^+^ current. In general, variations in its current amplitude and dynamic characteristics may affect several normal physiological functions of the heart, such as the contractile function and impulse conduction process to potentially cause arrhythmias (Workman et al., 2001). Amiodarone, a commonly prescribed medication for arrhythmia, exerts its effects by inhibiting potassium channels, which results in prolonging the third phase of the action potential in the heart’s conduction system cells (Zimetbaum, 2007; Lei et al., 2018).

Multiple mechanisms have been hypothesized to contribute to AF development *via* structural and electrical remodeling of the atrial tissue. Several factors, including multiple wavelets and increased focal autoregulation in the atria, have been intensively investigated, and their importance during this process has been confirmed (Sagris et al., 2021). Variations in the electrical remodeling of cardiac ion channels are associated with AF. Early and delayed afterdepolarizations may underlie the ectopic activity that triggers AF (Brundel et al., 2022). Since the phase 0 depolarization generated by the fast sodium channel is rapid and of short duration, it has minimal impact on the tricuspid effective refractory period (ERP). Therefore, the shortening of the ERP occurs primarily during the repolarization phase, particularly in phases 2 and 3. The physiological structures within the atrium, including the pulmonary vein muscle cuffs, valves, superior and inferior vena cava inlets, and coronary sinuses, could all be the anatomical basis for fold formation.

Manville et al. demonstrated that ALO relaxes blood vessels by activating the Kv7.5 vascular-expressed potassium channel (Manville et al., 2019). However, the effect of ALO on cardiac Kv channels remains unclear. Kv4 channels represent the primary subtypes of transient outward currents, characterized by rapid activation and inactivation. They play a crucial role in shaping the morphology of myocardial action potential (AP).

In this study, we investigated the impact of ALO on I_to_ in rat atrial myocytes *in vitro* and assessed its influence on acetylcholine-induced arrhythmias *in vivo*. Molecular docking was performed to validate ligand-protein binding interactions. This provides a preliminary theoretical and practical basis for the further development and utilization of this drug and its analog compounds.

## 2 Materials and methods

### 2.1 Animals

Male and female SD rats (190–200 g) were procured from Yangzhou University Comparative Medical Center (SCXK [Su] 2017-0044). The rats were caged in a thermoregulated room at 23°C ± 2°C with a relative humidity of 55% ± 5%. Animal protocols were conducted according to the guidelines of the Institutional Animal Care and Ethics Committee of Yangzhou University, which are aligned with international standards for research ethics and integrity. All experimental procedures were approved by the institution.

### 2.2 Drugs and reagents

Aconitine and ALO were provided by Chengdu EFA Biotechnology Co., Ltd. (Chengdu, China). Collagenase type II (batch number: 44N15308B) and bovine serum albumin (BSA, batch number: 8398F) were purchased from Worthington Co. (NJ, United States) and ICN (CA, United States). CsCl (batch number: 101368097), TEA.Cl (batch number: 101394223), taurine (batch number: 101282014T), MgATP (batch number: 1001571838), and HEPES (batch number: 1001621577) were acquired from Sigma-Aldrich (St. Louis, MO, United States). Heparin sodium (batch number: 301A028) and glucose (batch number: 405A096) were obtained from Solarbio Company (Beijing, China). Egtazic acid (EGTA, batch number: 101257840) and tetrodotoxin (TTX, batch number: AF02) were purchased from Sigma-Fluka Company (St. Louis, MO, United States) and AFFIX Company (Kfar Yona, Israel), respectively. All other reagents were of analytical grade.

### 2.3 Single cell isolation

Isolation of single atrial myocytes from SD rat hearts was performed as previously described (Guinamard et al., 2014). Briefly, the animals were heparinized (2000 IU/kg, i.p.) for 10 min and then anesthetized with 2% sodium pentobarbital solution (40 mg/kg, i.p.). Following successful anesthesia, the animals were placed in the supine position to open the chest. The heart was cut and then cleaned in Ca^2+^-free Tyrode’s solution containing the following compounds (in mM): HEPES 5.0, glucose 10.0, NaH_2_PO_4_ 0.33, MgCl_2_ 1.0, KCl 5.4, and NaCl 135 (pH adjusted to 7.3 with NaOH). Excess tissue was trimmed to ligate the aorta into the Langendorff perfusion device. The calcium-saturated Tyrode’s solution was obtained by adding CaCl_2_ (1.8 mM) to the Ca^2+^-free Tyrode’s solution to resuscitate the cardiac tissue for 30 s and to remove the remaining blood. Residual blood from the heart was washed away after 5 min of continuous perfusion with Ca^2+^-free Tyrode’s solution. The hearts were then digested with Ca^2+^-free Tyrode’s solution containing taurine (0.4 g/L), BSA (1 g/L), and type II collagenase (0.4 g/L) for 20 min until the heart became enlarged and soft, and the effluent became viscous and turbid. Temperature was maintained at 37°C during perfusion with 95% O_2_ and 5% CO_2_. The atrial tissue was cut and filtered using a 100-mesh filter to obtain individual atrial myocytes, and the cell suspension was washed three times with Krebs solution containing (in mM): EGTA 1.0, KCl 25.0, KOH 89.0, glucose 11.0, taurine 10.0, L-glutamic acid 70.0, NaH_2_PO_4_ 10.0, and HEPES 5.0 (pH adjusted to 7.4 with KOH) for 10 min to discard the supernatant. The cells were maintained at room temperature (23°C ± 2°C) for 30 min in the dark and then stored in Krebs solution at 4°C.

### 2.4 Whole-cell patch-clamp recording

Cell suspensions were transferred to a Petri dish containing the bath solution for recording I_to_ with Ca^2+^-saturated Tyrode’s solution. Once the cells adhered to the dish, those exhibiting favorable characteristics (elongated shape, smooth edges, intact, distinct striations, strong stereoscopic sense, and absence of contraction) were chosen for subsequent experiments under an IX73 Inverted Microscope (OLYMPUS Company, Nagano, Japan). Glass microelectrodes were constructed with borosilicate glass electrodes (1.50 mm outer diameter, 1.14 mm inner diameter) from Wuhan Microprobe Co., Ltd. (Wuhan, China) using a P-97 microelectrode puller (Sutter Co, California, United States). The pipette solution used for recording I_to_ included had a resistance of 1–3 MΩ and was comprised of the following (in mM): KCl 40.0, K-Asparate 106.0, MgCl_2_ 1.0, EGTA 10.0, HEPES 5.0, and K_2_ATP 5.0 (pH adjusted to 7.3 with KOH). The EPC-10 USB/Patchmaster Single Channel Patch Clamp Amplifier (HEKA, Reuttlingen, Germany) and MP-225 Micromanipulator System (Sutter Co, California, United States) were used to record the whole-cell currents. Patchmaster software was utilized for the voltage clamp protocol generation, data acquisition, and current traces analysis. All of the experiments were conducted at room temperature (22°C–25°C).

### 2.5 Establishment of an *in vivo* model of AF

Twenty-four SD rats were randomly assigned to four groups that included control (*n* = 6), model (*n* = 6), amiodarone (*n* = 6), and 10 mg ALO (*n* = 6). The rat tails were soaked in 40°C warm water for approximately 3 min to soften the corneal layer and facilitate puncture. In addition to the control group, the other three groups of rat tails were disinfected with 75% ethanol solution, and a mixture of calcium chloride (10 mg/mL) and acetylcholine (66 μg/mL) was injected into the tail vein (1 mL/kg·d) for 10 days to establish an AF model. The rats in the control group were intraperitoneally injected with an equal dose of a 0.9% NaCl solution. From the fourth day of modeling, rats in the amiodarone group and the ALO group were intravenously injected with a mixture of CaCl_2_-Ach solution 1 hour before, receiving intravenous injections of amiodarone at a dose of 15 mg/kg and ALO at a dose of 10 mg/kg once daily for 7 consecutive days. The control group and model group rats were intravenously injected with an equal volume of 0.9% NaCl solution via the tail vein. The rats were injected intraperitoneally with 10% chloral hydrate solution (0.38 g/kg) for anesthesia. The RM6240 Biological Data Acquisition and Analysis System (Chengdu Instrument Factory, Chengdu, China) was utilized for analyzing electrocardiograms (ECGs) from standard limb lead II.

### 2.6 Molecular docking

The crystal structures of *KCND2* (PDB ID: 1NN7 Chain A), KCND3 (PDB ID: 2I2R Chain A), and *KCNIP4* (predicted protein crystal structure generated by AlphaFold2) were acquired from the RCSB PDB database or AlphaFold2. The acquired protein crystals were processed using the Protein Preparation Wizard module in Schrödinger software. The 2D sdf structure files of ALO and amiodarone obtained from the PubChem database (https://pubchem.ncbi.nlm.nih.gov/) were processed using the LigPrep module in the Schrödinger software to generate their respective 3D chiral conformations. The SiteMap module in the Schrödinger software was employed to predict the optimal binding sites between the ligand and protein. Thereafter, the Receptor Grid Generation module in the Schrödinger software was used to set the most appropriate enclosing box to wrap the predicted binding sites perfectly, and the active sites of the three proteins were obtained on this basis. Molecular docking (with the highest precision, XP docking) was performed on the two ligand compounds and the active sites of three proteins. The interaction of the ligand and the active sites of the protein was analyzed through the MM-GBSA calculation. MM-GBSA dG Bind can approximately represent the binding free energy between ligands and proteins.

### 2.7 Data analysis

Data are presented as mean ± standard deviation (SD). Curve fitting was performed using GraphPad Prism 6 software (Axon Instruments). The experimental data were analyzed using Origin 7.0 (Microcal Software, Northampton, MA, United States). Statistical significance was assessed using SPSS 25.0 (SPSS, Inc., Chicago, IL, United States). The *t*-test was employed for comparing two groups, whereas analysis of variance (ANOVA) was utilized for comparing multiple groups. A value for *P* of less than 0.05 was considered to be statistically significance. The study is explorative in character, and as such, *p*-values need to be considered as descriptive and provisional.

## 3 Results

### 3.1 Effect of ALO on peak I_to_ in atrial myocytes

**FIGURE 1:**
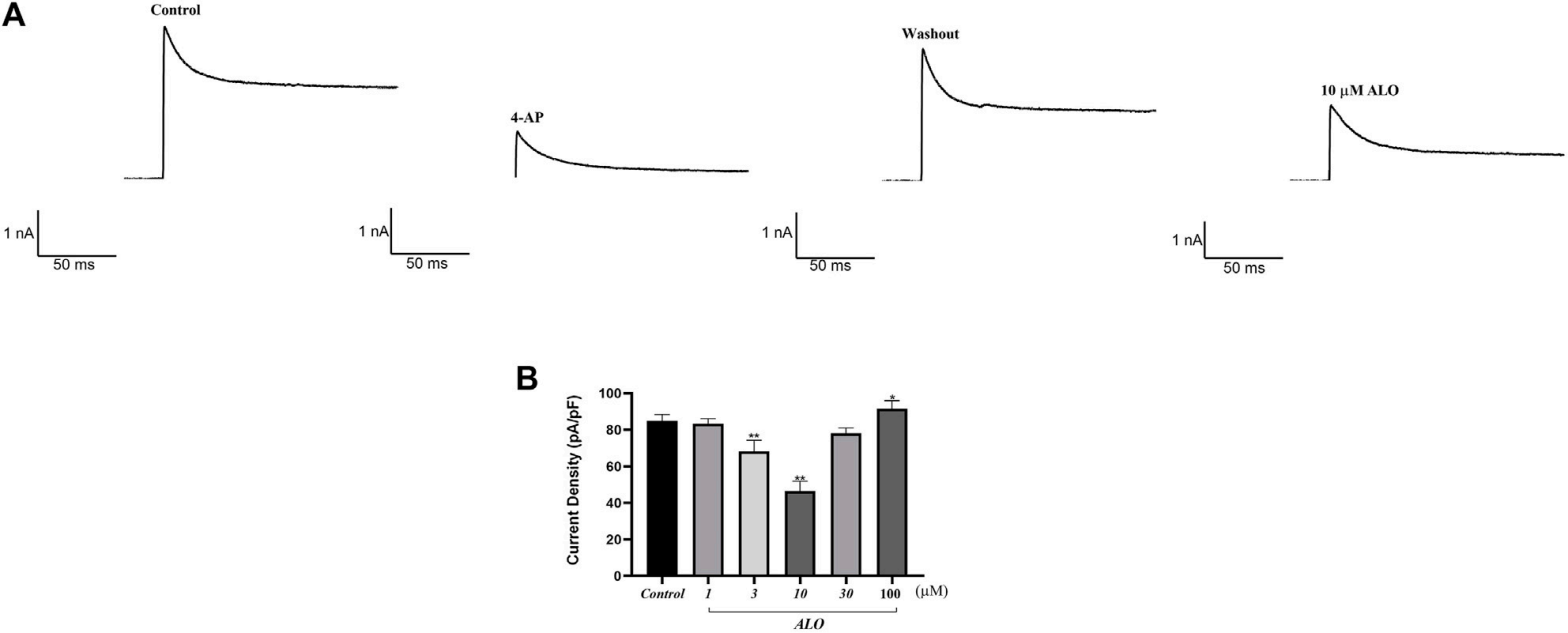
Effect of ALO on I_to_ in atrial myocytes (data from double determinations in *n* = 3 rats, representing a total of *n* = 6 measurements). **(A)** Effect of ALO on the original current for the single stimulus diagram of I_to_. **(B)** The effects of different concentrations of ALO on I_to_. *p < 0.05, **p < 0.01 *versus* the control.

To examine the impact of ALO on peak I_to_, a single square-wave stimulus of 70 mV for 300 ms at a holding potential of −80 mV was administered in the voltage clamp mode to elicit an outward current. As presented in Figure 1A, the current was almost completely suppressed by 2 μM 4-aminopyridine (4-AP), an I_to_-specific blocker, and it was partially restored by removing 4-AP. The current induced in this stimulation mode was I_to_. Subsequently, 10 μM ALO was perfused to inhibit I_to_; however, the inhibitory effect was not as significant as that of 4-AP. I_to_ induced by ALO at various concentrations is depicted in Figure 1B. We noted that 1 μM ALO elicited no significant alterations compared to the control group, whereas treatments with 3 and 10 μM ALO resulted in a reduction relative to the control group. This observation suggests that 3 and 10 μM ALO can inhibit transient outward potassium channels. Moreover, the current density was slightly higher than that of the control group when the concentration was >30 μM. An increase in I_to_ is the mechanism that causes Brugada syndrome and early repolarization syndrome. Nevertheless, excessive inhibition or an increase in I_to_ may induce abnormal changes in the channel, ultimately resulting in cardiovascular disease. ALO at 3 and 10 μM moderately affected I_to_ and alleviated the abnormal function and expression of I_to_ in the diseased hearts. Therefore, 3 and 10 μM ALO were used for subsequent experiments. As presented in Table 1, the peak current is displayed as the current density (pA/pF). At 70 mV depolarization voltage, the concentration of ALO at 1, 3, and 10 μM suppressed I_to_ and reduced the current by 3.03% ± 3.65%, 16.92% ± 5.16%, and 30.09% ± 6.13% (*p* < 0.01), respectively.

**TABLE 1:** Peak current density measured at 70 mV for various concentrations of ALO.

Group (concentration)	Current density (pA/pF)
Control	85.80 ± 3.59
1 µM ALO	80.64 ± 3.65
3 µM ALO	68.38 ± 4.12**
10 µM ALO	45.75 ± 5.32**

### 3.2 Effect of ALO on the I-V curve of I_to_ channels

**FIGURE 2:**
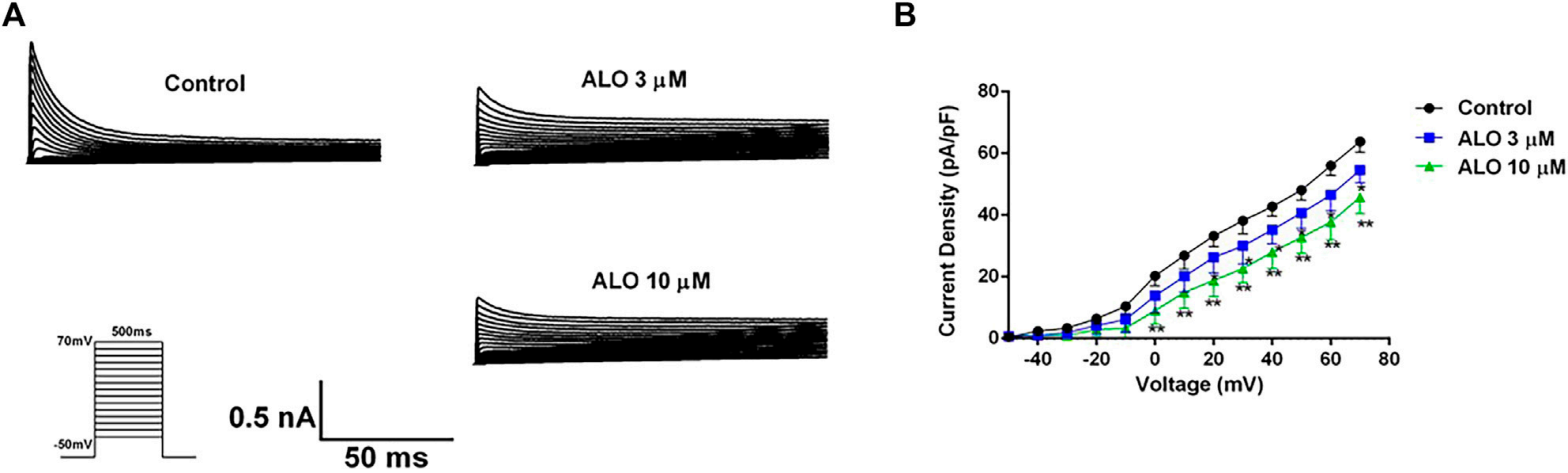
I-V curve recordings of I_to_ (data from double determinations in *n* = 3 rats, representing a total of *n* = 6 measurements). **(A)** I-V traces recorded before and after the administration of ALO. **(B)** Effects of ALO on I_to_ current density under different voltages. Values are presented as mean ± SD. *p < 0.05, **p < 0.01 vs. the control.

**FIGURE 3:**
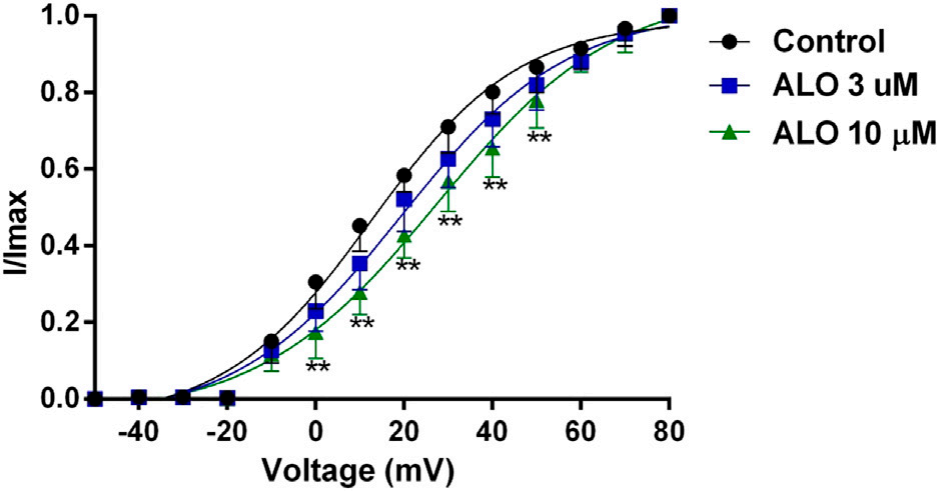
Effects of ALO on the activation gated-kinetic characteristics of I_to_ (data from double determinations in *n* = 3 rats, representing a total of *n* = 6 measurements). Values are presented as mean ± SDs (*n* = 6). *p < 0.05, **p < 0.01 vs. the control.

To investigate the impact of ALO on I_to_ across various voltage conditions, we maintained the potential at −80 mV in the voltage clamp mode. We applied a square wave ranging from −50–70 mV in 10 mV increments for 500 ms at 0.5 Hz to elicit I_to_ response before and after ALO administration. As presented in Figures 2A, 3 and 10 μM ALO treatments reduced the current compared to the control group. Figure 2B indicates that 3 and 10 μM ALO treatments significantly shifted the I-V curve down without changes in the curve shape. Specifically, the curve moved toward the positive direction of the current axis, with the current density exhibiting a significant increase at different membrane potentials (particularly at 70 mV).

### 3.3 Effect of ALO on I_to_ activation curves

To investigate the relationship between the inhibitory effect of ALO on I_to_ and the activation state of voltage-gated potassium channels, we fitted the activation curve based on the relevant data from the I-V curves. This allowed us to assess the impact of ALO on activation kinetics curves. As presented in Figure 3, various concentrations of ALO induced a shift in the activation curve of I_to_ in a positive direction. Particularly, the administration of 3 and 10 μM ALO in an increase in the V_1/2-ac_ value from 12.58 ± 1.70 mV (pre-administration) to 19.48 ± 1.74 and 37.55 ± 1.67 mV (data from double determinations in n = 3 rats, representing a total of *n* = 6 measurements; *p* < 0.01 vs. control), respectively. Furthermore, the slope (κ) values were 16.73 ± 1.76 (pre-administration), 18.79 ± 1.88 (data from double determinations in *n* = 3 rats, representing a total of *n* = 6 measurements; *p* > 0.05 vs. control), and 30.17 ± 1.72 (data from double determinations in *n* = 3 rats, representing a total of n = 6 measurements; *p* < 0.01 vs. control). Consequently, ALO slowed the activation of I_to_, thus blocking the opening of the potassium channel.

### 3.4 Effect of ALO on I_to_ inactivation curves

**FIGURE 4:**
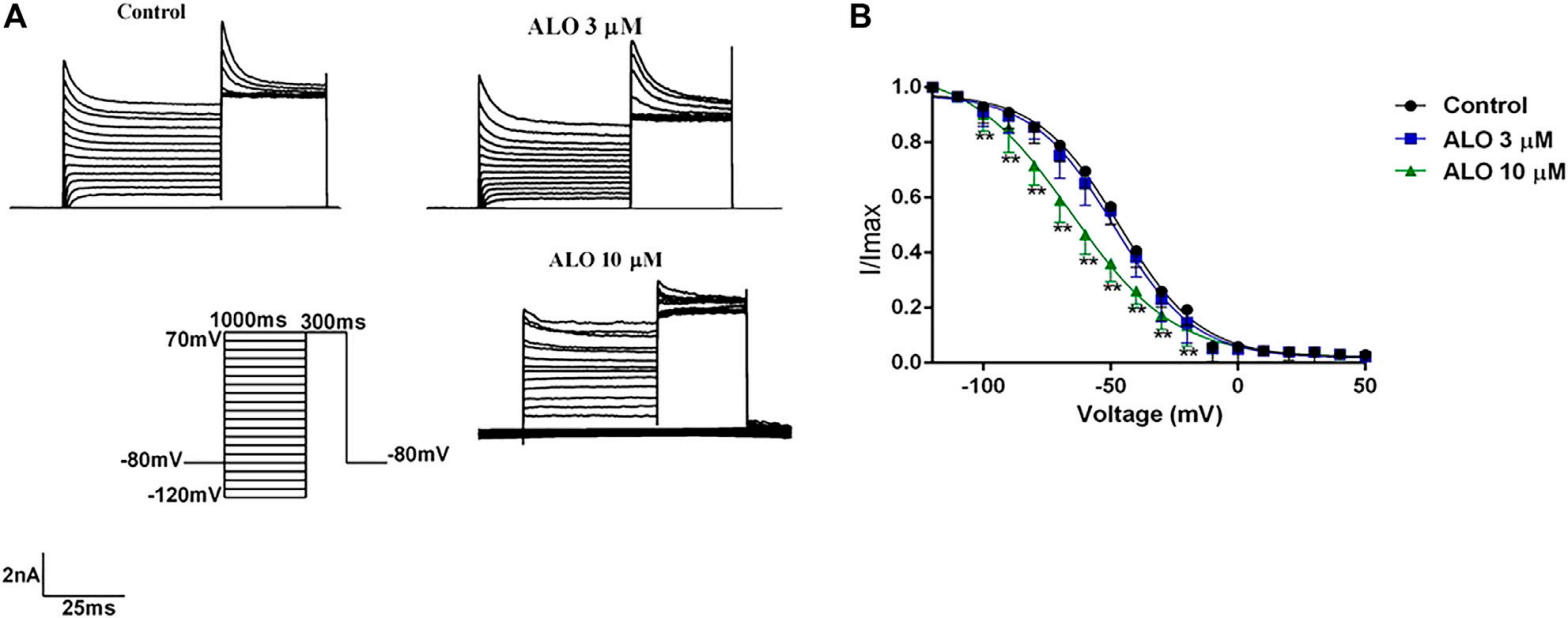
Effects of ALO before and after administration on the inactivation curve (data from double determinations in *n* = 3 rats, representing a total of *n* = 6 measurements). **(A)** Original traces of I_to_ recorded in the presence or absence of ALO. **(B)** Steady-state inactivation dynamic characteristics for control and after 3 and 10 μM ALO treatments. Data are provided as the mean ± SDs (*n* = 6). *p < 0.05, *p < 0.01 vs. the control.

To test the relationship between ALO and the inactivation state in the gating dynamics characteristics, the stimulus protocols were as follows: the condition pulse was gradually depolarized from −120 mV to 30 mV in 10 mV increments for 1,000 ms at a holding potential of −80 mV, and a test pulse was set to 70 mV for 300 ms. The currents before and after ALO administration were induced by the double-pulse stimulation (Figure 4A). The steady-state inactivation curve was fitted using the Boltzmann equation $\left(I/I_{\max}=1/\left[1+\exp^{\left\{\left(V-V_{1/2}\right)/K\right\}}\right]\;;\text{ Eq}.\;1\right)$ , where V_1/2_ indicates the potential corresponding to the half-maximal inactivation point and κ represents the slope factor. As presented in Figure 4B, 10 μM ALO significantly shifted the inactivation curve of I_to_ to the left in the direction of hyperpolarization, whereas 3 μM ALO resulted in no obvious changes compared to the control group. The V_1/2-in_ values before and after the administration of ALO were changed from −46.62 ± 1.94 mV (pre-administration) to −48.74 ± 1.09 mV (data from double determinations in *n* = 3 rats, representing a total of n = 6 measurements; *p* > 0.05 vs. control) and −65.56 ± 1.02 mV (data from double determinations in *n* = 3 rats, representing a total of *n* = 6 measurements; *p* < 0.01 vs. control), respectively. Moreover, the κ values were 15.86 ± 0.87 (pre-administration), 15.90 ± 1.07 (data from double determinations in *n* = 3 rats, representing a total of *n* = 6 measurements; *p* > 0.05 vs. control), and 29.83 ± 1.97 (data from double determinations in *n* = 3 rats, representing a total of *n* = 6 measurements; *p* < 0.01 vs. control), respectively. These results reveal that the steady-state inactivation process was only affected by 10 μM ALO.

### 3.5 Effects of ALO on the recovery curve after inactivation of I_to_

**FIGURE 5:**
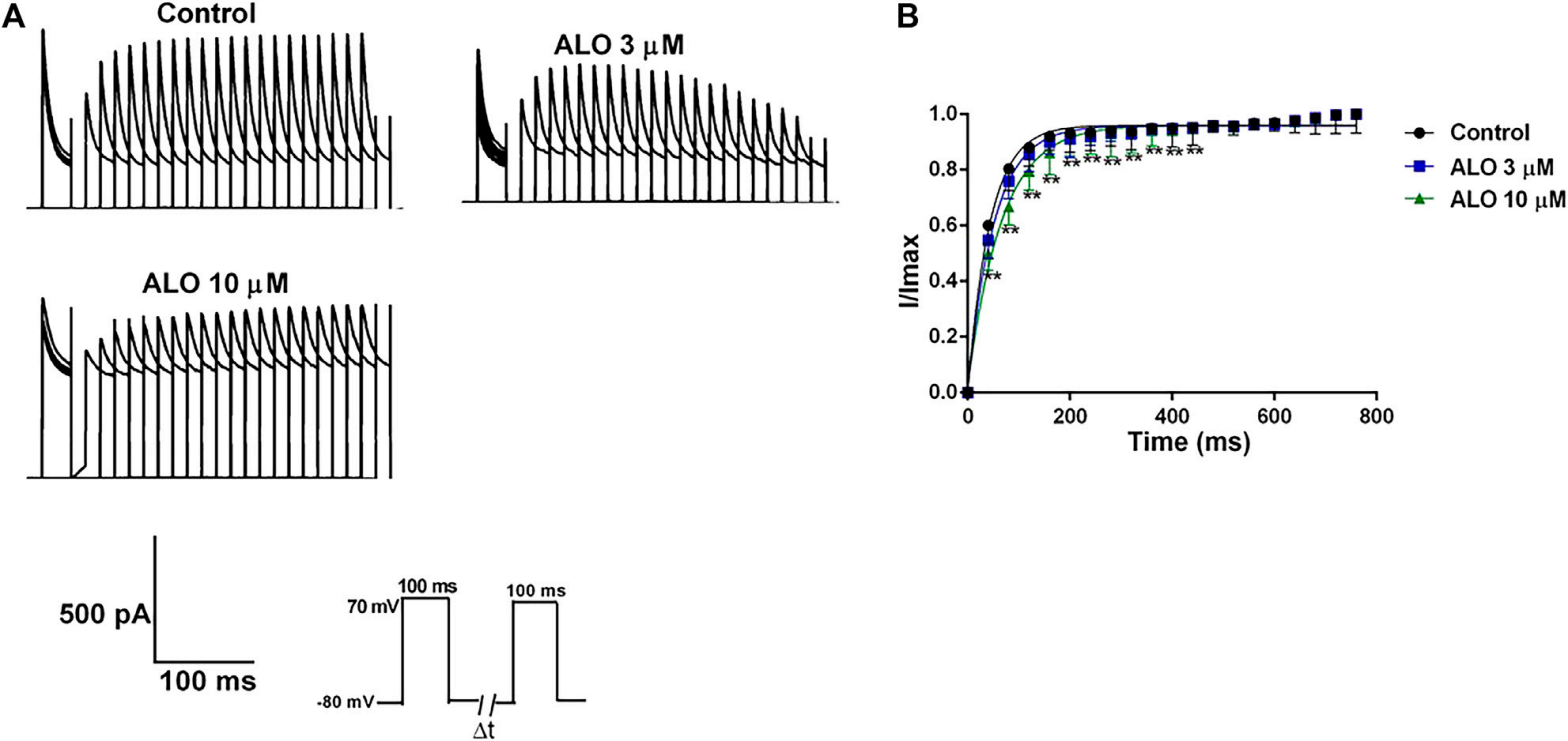
Effects of ALO on the I_to_ recovery curve after inactivation in atrial myocytes (data from double determinations in *n* = 3 rats, representing a total of *n* = 6 measurements). **(A)** Original traces in control and in response to 3 or 10 μM ALO. **(B)** Kinetic characteristics of the recovery curve after inactivation were analyzed in both control conditions and in response to ALO (3 or 10 μM). Values are presented as mean ± SD. *p < 0.05, **p < 0.01 vs. the control.

The steady-state recovery curves after inactivation were acquired *via* the double pulse voltage protocol as follows: Initially, a holding potential of −80 mV was maintained, followed by a depolarization to 70 mV stimulus for 200 ms. Subsequently, repolarization to 70 mV for 200 ms was conducted, and the interval time between two pulses was gradually increased by 50 ms for a total of 20 pulses. The original current of the curve before and after ALO administration was documented (Figure 5A), and it is provided in Figure 5B. The steady-state recovery curves after inactivation of I_to_ were fitted by the one-phase association equation described as $I/I_{\max}=1\;–\exp\left(-t/\tau \right)\;\left(\text{Eq}.\;2\right)$ , where t represents the time interval between two pulses and τ is the time constant of reactivation after inactivation. Thereafter, the τ value was changed from 43.51 ± 3.06 ms under control conditions to 56.73 ± 3.03 ms in response to 3 μM ALO (n = 6; *p* < 0.05 vs. control) and 65.93 ± 3.55 ms in response to 10 μM ALO (*n* = 6; *p* < 0.01 vs. control). The results indicated that 3 and 10 μM ALO significantly inhibited I_to_ by affecting the recovery kinetic characteristics after inactivation and prolonging the recovery time after inactivation.

### 3.6 Effect of ALO on the ECG of rats with AF

**FIGURE 6:**
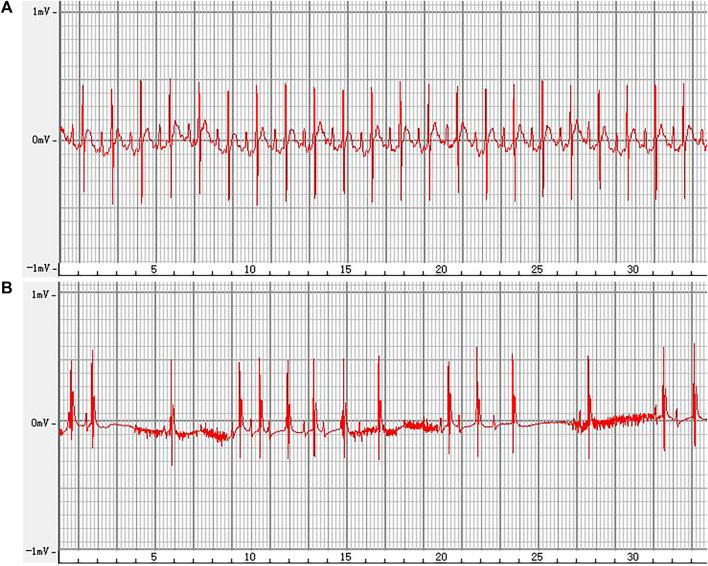
Representative ECG recordings, from a normal rat **(A)**, and of an AF rat **(B)**.

The representative ECGs in rats are presented in Figure 6. The control group exhibited a normal ECG. Varying RR intervals, the appearance of f waves, and the disappearance of p waves are considered indicators of AF onset, while the disappearance of f waves and the appearance of p waves are considered indicators of AF termination. AF models were successfully established in other groups. The induction rate of AF in model group is greater than 80%. Compared to the model group, pretreatment with amiodarone and ALO prolonged the induction time of AF and reduced the duration of AF in the animal models, as shown in Table 2.

**TABLE 2:** Induction and duration time of AF among rats in each group.

Group	Induction time of AF/s	Duration of AF/s
control	-	-
model	4.64 ± 1.25	7.64 ± 1.05
amiodarone	8.65 ± 1.13*	3.25 ± 1.02*
ALO	6.75 ± 1.32*	5.73 ± 0.87*

*p < 0.05 vs. the model.

### 3.7 Docking results

**FIGURE 7:**
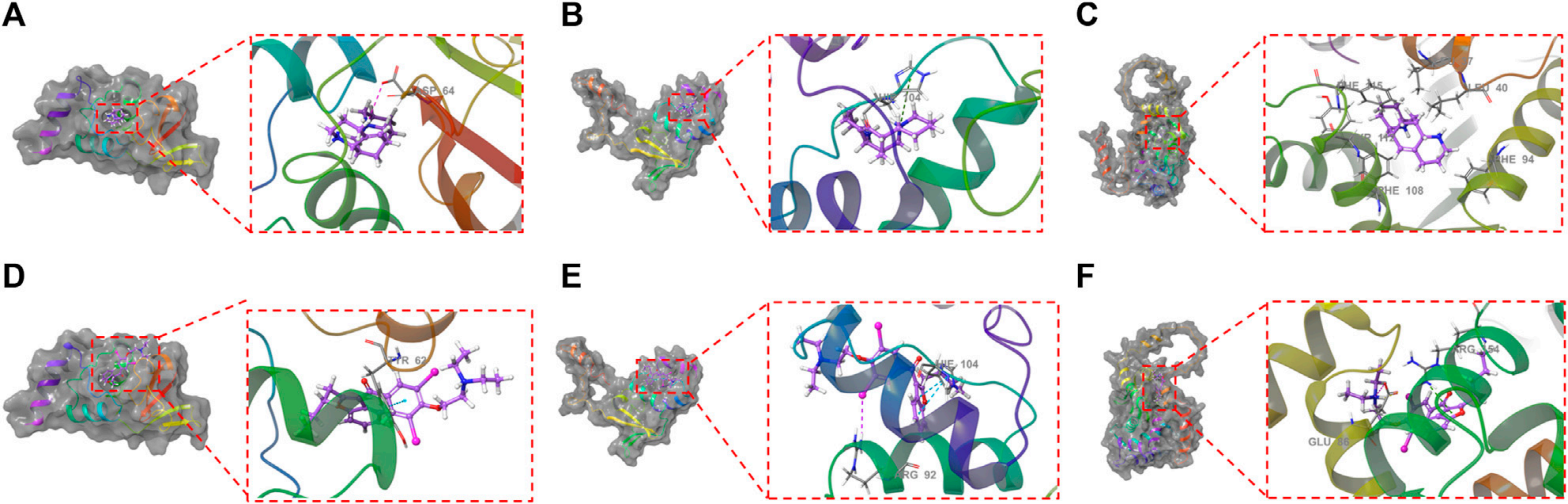
Molecular docking between ALO and the three proteins (KCND2, KCND3, and KCNIP4) based on crystal structures: **(A)**. The ligand forms a salt bridge with the residue ASP64; **(B)**. The ligand forms a π-cation bond with the residue HIE104; **(C)**. KCNIP4. Molecular docking between amiodarone and the three proteins (KCND2, KCND3, and KCNIP4) based on crystal structures: **(D)**. The ligand forms a π-π bond with the residue TYR62; **(E)**. The ligand forms two π-π bonds with the residue HIE104 and a halogen bond with the residue ARG92; **(F)**. The ligand forms a π-cation bond with the residue ARG154 and a hydrogen bond with a salt bridge with the GLU86 residue.

XP docking results should be interpreted based on the XP Gscore. Typically, a value below −6 is considered indicative of stable binding performance between the ligand and the protein. MM-GBSA analysis results should be referred to as MM-GBSA dG Bind, and when this value is below −30 kcal/mol, it suggests low binding free energy and indicates a stable binding between the ligand and the protein. The docking score of ALO with KCND2 is −3.990, and the MM-GBSA dG Bind result is −30.68 kcal/mol, thus indicating stable binding. In contrast, the docking scores of ALO with KCND3 and KCNIP4 are −2.106 and −5.339, with MM-GBSA dG Bind results of −10.67 and −7.2 kcal/mol, respectively. The higher binding free energy suggests fewer stable interactions of ALO with these two proteins. Additionally, the docking score of amiodarone with KCND2 is −1.75, and the MM-GBSA result is −35.26 kcal/mol. Despite the low binding free energy, the high docking score suggests a low likelihood of binding between amiodarone and KCND2, but the stability after binding may still be substantial. The docking scores of amiodarone with KCND3 and KCNIP4 are −5.363 and −3.004, respectively, with MM-GBSA results of −23.67 and −27.66 kcal/mol. Both the binding free energy and docking scores are relatively low, thus indicating a stable binding of amiodarone with these two proteins. The results of the molecular docking assessments are presented in Table 3 and Figure 7.

**TABLE 3:** Molecular docking results.

Compound	Target	XP Gscore	MM-GBSA dG Bind(kcal/mol)	Hydrophobic interaction
ALO	KCND2	−3.990	−30.68	ILE128, PRO126, ILE125, LEU124
Amiodarone		−1.750	−35.26	LEU124、ILE125、PRO126、ILE128、PRO63
ALO	KCND3	−2.106	−10.67	TYR105, PRO106, CYS132
Amiodarone		−5.363	−23.67	TYR108、TYR132
ALO	KCNIP4	−5.339	−7.20	PHE115, TYR112, PHE108, PHE94, LEU40
Amiodarone		−3.004	−27.66	LEU41、LEU153、MET38

## 4 Discussion

AF often leads to high disability and mortality rates, as well as complications such as stroke and heart failure, imposing significant economic burdens on society (Varró et al., 2021). It is currently a serious cardiovascular disease that seriously endangers public health. Kv channels, crucial for cardiac electrical activity, primarily influence the repolarization process of AP and contribute significantly to excitation-contraction coupling and arrhythmias (Zhang et al., 2006; Amin et al., 2010). Inhibiting I_to_ can prolong APD, reduce ventricular prematureness, halt phase 2 re-entry, and ultimately prevent the occurrence of ventricular tachycardia and fibrillation (Marquez et al., 2007). Moreover, alterations in the current amplitude and dynamic properties of I_to_ indirectly influence the activation and inactivation of other ion channels, consequently impacting the AP shape, APD, and ERP. Therefore, I_to_ is an important current that controls the AP in myocardial cells. Repolarization is governed by a delicate balance of various inward and outward transmembrane ionic currents, along with electrogenic pump mechanisms. Repolarization reserve plays a crucial role in elucidating the pathogenesis of arrhythmias stemming from repolarization abnormalities and drug-induced proarrhythmic adverse effects (Varró and Baczkó, 2011; Virág et al., 2011). Understanding the impact of I_to_ inhibition on patients with impaired repolarization reserve is essential for elucidating the mechanisms underlying arrhythmias and guiding potential therapeutic interventions. In a study, inhibiting I_to_ in cases of impaired repolarization reserve may lead to excessive prolongation of repolarization, resulting in the formation of early afterdepolarizations, thereby increasing the risk of arrhythmias (Varró and Baczkó, 2011). An increase in I_to_ is the mechanism that causes Brugada syndrome and early repolarization syndrome. Excessive inhibition or an increase in I_to_ may induce abnormal changes in the channel, ultimately resulting in cardiovascular disease. In our experiment, we utilized 3 and 10 μM ALO, without employing higher concentrations. In our current study, 10 µM of ALO inhibited I_to_, suggesting its ability to block transient outward potassium channels. However, as the concentration increased to 30 and 100 μM, we noted an increasing trend in I_to_ (pA/pF). This result may stem from the complex mechanisms of action of ALO on ion channels. At higher doses, ALO may interact with other cellular components or pathways that counteract its effects on I_to_, or it may produce other effects on cellular electrophysiology. This change may be attributed to the concentration-dependent effects of ALO, where its selectivity or affinity for ion channels may vary at different concentrations. Additionally, another possibility is that at higher concentrations, ALO may saturate its target binding sites, leading to a plateau effect on I_to_ inhibition. The combined effects of these factors may result in the observed increasing trend in I_to_ at concentrations of 30 and 100 µM. Further research is needed to elucidate the detailed molecular mechanisms.

Our data indicate that 3 and 10 μM ALO treatments inhibited the current density of I_to_. The gated kinetics analyses revealed that ALO decreased the I_to_ current density, possibly by decelerating the steady-state activation process of the channel and shifting the half-activation voltage to a positive value. This indicates a reduction in the opening of the channel under the same depolarization voltage stimulation. Additionally, ALO accelerated the inactivation of I_to_, shifted the inactivation curve to the left, and extended the recovery time after steady-state inactivation. This caused the recovery curve to move to the right, and this slowed down the recovery process from the inactive state. Hence, the antiarrhythmic mechanism of ALO might be elucidated by its inhibition of I_to_ in a concentration-dependent manner, which impacts the processes of steady-state activation, inactivation, and recovery after inactivation.

Acetylcholine exposure increases the opening of the acetylcholine-sensitive potassium channel (IK-Ach) in the atrium, leading to a notable enhancement in the potassium outflow current intensity. This results in the shortening of atrial repolarization time and action potential duration (APD), subsequently shortening the effective inactivity period, and contributing to fold formation, and further promoting AF (Moss et al., 2018). Moreover, acetylcholine can diminish sinoatrial node autoregulation by activating M receptors, resulting in a relative increase in atrial potential pacing point autoregulation, thereby contributing to folding and AF formation. Calcium chloride increases inward Ca^2+^ flow during the phase 2 plateau, thus leading to shortened ERP and APD and facilitating easier maintenance of AF (Li et al., 2018; Fan et al., 2023). The administration of ALO prolonged the induction time of AF and shortened the duration of AF in rats. The results for the *in vivo* ECG parameters revealed that ALO may prevent acetylcholine-induced AF in rats. The principle of clinical drug administration is to administer the drug as sparingly as possible to achieve therapeutic effects, ensuring optimal dosage, maximizing its protective effects, and minimizing side effects. The blood concentration of a drug serves as a measure of its concentration in the body and is directly linked to the efficacy and toxicity of the drug. A LC-MS/MS method was effectively developed and validated to quantitatively measure ALO in rat plasma with speed and sensitivity, demonstrating its suitability for investigating ALO pharmacokinetics. The method unveiled a relatively high oral bioavailability of 44.87% following both oral and intravenous administrations (Huang et al., 2021).

Although the mechanism of AF induced by calcium chloride and acetylcholine is clearly understood, additional arrhythmias and abnormal electrocardiographic phenomena have been observed in AF animal models. Further studies are required to determine if calcium chloride and acetylcholine play a role in these processes (Bayer et al., 2019). In addition to atria, calcium chloride and acetylcholine can act on the ventricular muscle, particularly calcium chloride (Murray et al., 2022). The transient atrioventricular block after administration that lasts <1 min is associated with an atrioventricular conduction disturbance caused by acetylcholine that disappears naturally after acetylcholine is hemodiluted and metabolized. Overloaded intracellular calcium leads to myocardial cell damage resulting in conduction disturbances in the ventricular myocardium, delayed post-depolarization leading to increased autoregulation at ectopic rhythm points, and increased Ca^2+^ inward flow that leads to shortened ERP and APD and forms the basis for all types of ventricular tachycardia (Wleklinski et al., 2020). Myocardial conduction disturbances results in heterogeneous myocardial repolarization and increased dispersion of myocardial repolarization across the wall, which may be associated with QRS-T-wave electrical alternations. Polymorphic ventricular premature beats can occur due to increased autoregulation and shortened effective inactivity at ectopic rhythm points. When ventricular premature beats occur late, the atria cannot be excited by retrograde transmission, but can be excited by the sinoatrial node. As a result, normal P waves may be observed earlier in the ventricle.

Kv4.2 (encoded by *KCND2*) is a predominant transient outward potassium channel. In the molecular docking stimulation, ALO binds to the surface of the active pocket of the *KCND2* protein, forming hydrophobic interactions with residues ILE128, PRO126, ILE125, and LEU124 of the Kcnd2 protein. Additionally, this ligand formed a salt bridge with residue ASP64. ALO binds to the active pocket surfaces of both KCND3 and KCNIP4 proteins, but the interactions are relatively unstable. The results of molecular docking experiments revealed that ALO could stably bind to the KCND2 protein. These results provide a rational explanation for how ALO affects cardiomyocyte I_to_ gating kinetics.

However, it is important to acknowledge the limitations of our study. Cardiac potassium channels are classified into transient outward, delayed rectifier outward, and inward rectifier currents. Our investigation primarily focused on the inhibitory effects of ALO on I_to_ in rat ventricular myocytes and did not explore its potential effects on other potassium ion channels or cardiac electrophysiological parameters. Future comprehensive studies, including both basic research and clinical trials, are needed to provide more robust evidence.

In conclusion, ALO regulates I_to_ by reducing the current densities of I_to_ and affecting the channel gating kinetic characteristics to stabilize the cardiac repolarization process and reduce the risk of arrhythmias.

## Data Availability

The original contributions presented in the study are included in the article/Supplementary material, further inquiries can be directed to the corresponding author.

## References

[B1] Amin A. S. Tan H. L. Wilde A. A. (2010). Cardiac ion channels in health and disease. Heart Rhythm 7 (1), 117–126. 10.1016/j.hrthm.2009.08.005 19875343

[B2] Bayer J. D. Boukens B. J. Krul S. P. J. Roney C. H. Driessen A. H. G. Berger W. R. (2019). Acetylcholine delays atrial activation to facilitate atrial fibrillation. Front. Physiol. 10, 1105. 10.3389/fphys.2019.01105 31551802 PMC6737394

[B3] Brundel B. Ai X. Hills M. T. Kuipers M. F. Lip G. Y. H. de Groot N. M. S. (2022). Atrial fibrillation. Nat. Rev. Dis. Prim. 8 (1), 21. 10.1038/s41572-022-00347-9 35393446

[B4] Drum B. M. Yuan C. Li L. Liu Q. Wordeman L. Santana L. F. (2016). Oxidative stress decreases microtubule growth and stability in ventricular myocytes. J. Mol. Cell Cardiol. 93, 32–43. 10.1016/j.yjmcc.2016.02.012 26902968 PMC4902331

[B5] Fan X. Feng K. Liu Y. Yang L. Zhao Y. Tian L. (2023). miR-135a regulates atrial fibrillation by targeting Smad3. Cardiovasc Ther. 2023, 8811996. 10.1155/2023/8811996 37187923 PMC10181910

[B6] Guinamard R. Hof T. Salle L. (2014). Current recordings at the single channel level in adult mammalian isolated cardiomyocytes. Methods Mol. Biol. 1183, 291–307. 10.1007/978-1-4939-1096-0_19 25023317

[B7] Huang S. Zhang Y. Zhang Y. Liu J. Liu Z. Wang X. (2021). Establishment of LC-MS/MS method for determination of aloperine in rat plasma and its application in preclinical pharmacokinetics. J. Chromatogr. B 1173, 122671. 10.1016/j.jchromb.2021.122671 33819795

[B8] Lei M. Wu L. Terrar D. A. Huang C. L. H. (2018). Modernized classification of cardiac antiarrhythmic drugs. Circulation 138 (17), 1879–1896. 10.1161/CIRCULATIONAHA.118.035455 30354657

[B9] Li M.-t. Du Y. Y. Zhong F. Wang J. R. Gu Y. W. Zhang Y. (2021). Inhibitory effects of aloperine on voltage-gated Na+ channels in rat ventricular myocytes. Naunyn-Schmiedeberg's Archives Pharmacol. 394 (7), 1579–1588. 10.1007/s00210-021-02076-4 33738513

[B10] Li W. Li Y. Zhao Y. Ren L. (2020b). The protective effects of aloperine against ox-LDL-induced endothelial dysfunction and inflammation in HUVECs. Artif. Cells Nanomed Biotechnol. 48 (1), 107–115. 10.1080/21691401.2019.1699816 31852304

[B11] Li Y. Song B. Xu C. (2018). Effects of Guanfu total base on Bcl-2 and Bax expression and correlation with atrial fibrillation. Hell. J. Cardiol. 59 (5), 274–278. 10.1016/j.hjc.2018.02.009 29501704

[B12] Li Y. Wang G. Liu J. Ouyang L. (2020a). Quinolizidine alkaloids derivatives from Sophora alopecuroides Linn: bioactivities, structure-activity relationships and preliminary molecular mechanisms. Eur. J. Med. Chem. 188, 111972. 10.1016/j.ejmech.2019.111972 31884408

[B13] Lv X. Q. Zou L. L. Tan J. L. Li H. Li J. R. Liu N. N. (2020). Aloperine inhibits hepatitis C virus entry into cells by disturbing internalisation from endocytosis to the membrane fusion process. Eur. J. Pharmacol. 883, 173323. 10.1016/j.ejphar.2020.173323 32622669

[B14] MacKinnon R. (1995). Pore loops: an emerging theme in ion channel structure. Neuron 14 (5), 889–892. 10.1016/0896-6273(95)90327-5 7538310

[B15] Manville R. W. van der Horst J. Redford K. E. Katz B. B. Jepps T. A. Abbott G. W. (2019). KCNQ5 activation is a unifying molecular mechanism shared by genetically and culturally diverse botanical hypotensive folk medicines. Proc. Natl. Acad. Sci. U. S. A. 116 (42), 21236–21245. 10.1073/pnas.1907511116 31570602 PMC6800379

[B16] Mao Q. Guo F. Liang X. Wu Y. Lu Y. (2019). Aloperine activates the PI3K/akt pathway and protects against coronary microembolisation-induced myocardial injury in rats. Pharmacology 104 (1-2), 90–97. 10.1159/000500761 31163448

[B17] Marquez M. F. Salica G. Hermosillo A. G. Pastelín G. Gómez-Flores J. Nava S. (2007). Ionic basis of pharmacological therapy in Brugada syndrome. J. Cardiovasc Electrophysiol. 18 (2), 234–240. 10.1111/j.1540-8167.2006.00681.x 17338775

[B18] Moss R. Sachse F. B. Moreno-Galindo E. G. Navarro-Polanco R. A. Tristani-Firouzi M. Seemann G. (2018). Modeling effects of voltage dependent properties of the cardiac muscarinic receptor on human sinus node function. PLoS Comput. Biol. 14 (10), e1006438. 10.1371/journal.pcbi.1006438 30303952 PMC6197694

[B19] Murray L. E. Burchett H. Chowdhury S. M. Haney A. L. Hassid M. Strelow J. R. (2022). Calcium chloride infusions are not associated with improved outcomes in neonates undergoing cardiac operations. Pediatr. Cardiol. 43 (2), 366–372. 10.1007/s00246-021-02730-x 34523025 PMC9462392

[B20] Sagris M. Vardas E. P. Theofilis P. Antonopoulos A. S. Oikonomou E. Tousoulis D. (2021). Atrial fibrillation: pathogenesis, predisposing factors, and genetics. Int. J. Mol. Sci. 23 (1), 6. 10.3390/ijms23010006 35008432 PMC8744894

[B21] Thiel G. Homann U. Plieth C. (1997). Ion channel activity during the action potential in Chara: new insights with new techniques. J. Exp. Bot. 48, 609–622. 10.1093/jxb/48.Special_Issue.609 21245235

[B22] Varró A. Baczkó I. (2011). Cardiac ventricular repolarization reserve: a principle for understanding drug-related proarrhythmic risk. Br. J. Pharmacol. 164 (1), 14–36. 10.1111/j.1476-5381.2011.01367.x 21545574 PMC3171857

[B23] Varró A. Tomek J. Nagy N. Virág L. Passini E. Rodriguez B. (2021). Cardiac transmembrane ion channels and action potentials: cellular physiology and arrhythmogenic behavior. Physiol. Rev. 101 (3), 1083–1176. 10.1152/physrev.00024.2019 33118864

[B24] Virág L. Jost N. Papp R. Koncz I. Kristóf A. Kohajda Z. (2011). Analysis of the contribution of Ito to repolarization in canine ventricular myocardium. Br. J. Pharmacol. 164 (1), 93–105. 10.1111/j.1476-5381.2011.01331.x 21410683 PMC3171863

[B25] Wang H. Yang S. Zhou H. Sun M. Du L. Wei M. (2015). Aloperine executes antitumor effects against multiple myeloma through dual apoptotic mechanisms. J. Hematol. Oncol. 8, 26. 10.1186/s13045-015-0120-x 25886453 PMC4377192

[B26] Wang R. Deng X. Gao Q. Wu X. Han L. Gao X. (2020). Sophora alopecuroides L.: an ethnopharmacological, phytochemical, and pharmacological review. J. Ethnopharmacol. 248, 112172. 10.1016/j.jep.2019.112172 31442619

[B27] Wleklinski M. J. Kannankeril P. J. Knollmann B. C. (2020). Molecular and tissue mechanisms of catecholaminergic polymorphic ventricular tachycardia. J. Physiol. 598 (14), 2817–2834. 10.1113/JP276757 32115705 PMC7699301

[B28] Workman A. J. Kane K. A. Rankin A. C. (2001). The contribution of ionic currents to changes in refractoriness of human atrial myocytes associated with chronic atrial fibrillation. Cardiovasc Res. 52 (2), 226–235. 10.1016/s0008-6363(01)00380-7 11684070

[B29] Xu J. Yang Y. (2009). Traditional Chinese medicine in the Chinese health care system. Health Policy 90 (2-3), 133–139. 10.1016/j.healthpol.2008.09.003 18947898 PMC7114631

[B30] Ye Y. Wang Y. Yang Y. Tao L. (2020). Aloperine suppresses LPS-induced macrophage activation through inhibiting the TLR4/NF-κB pathway. Inflamm. Res. 69 (4), 375–383. 10.1007/s00011-019-01313-0 32144444

[B31] Zhang Y. Liu Y. Wang T. Li B. Li H. Wang Z. (2006). Resveratrol, a natural ingredient of grape skin: antiarrhythmic efficacy and ionic mechanisms. Biochem. Biophys. Res. Commun. 340 (4), 1192–1199. 10.1016/j.bbrc.2005.12.124 16406237

[B32] Zhang Y. Tachtsidis G. Schob C. Koko M. Hedrich U. B. S. Lerche H. (2021). KCND2 variants associated with global developmental delay differentially impair Kv4.2 channel gating. Hum. Mol. Genet. 30 (23), 2300–2314. 10.1093/hmg/ddab192 34245260 PMC8600029

[B33] Zhou H. Li J. Sun F. Wang F. Li M. Dong Y. (2020). A review on recent advances in aloperine research: pharmacological activities and underlying biological mechanisms. Front. Pharmacol. 11, 538137. 10.3389/fphar.2020.538137 33536900 PMC7849205

[B34] Zimetbaum P. (2007). Amiodarone for atrial fibrillation. N. Engl. J. Med. 356 (9), 935–941. 10.1056/NEJMct065916 17329700

